# Integrated Analysis of the ETS Family in Melanoma Reveals a Regulatory Role of ETV7 in the Immune Microenvironment

**DOI:** 10.3389/fimmu.2020.612784

**Published:** 2020-12-23

**Authors:** Hui Qu, Hui Zhao, Xi Zhang, Yang Liu, Feng Li, Liyan Sun, Zewen Song

**Affiliations:** ^1^Department of Plastic Surgery, Shanxi Bethune Hospital, Shanxi Academy of Medical Sciences, Taiyuan, China; ^2^Department of Urology, The Affiliated Hospital of Weifang Medical University, Weifang, China; ^3^Department of Oncology, The Third Xiangya Hospital of Central South University, Changsha, China; ^4^Department of Pathology, The Third Xiangya Hospital of Central South University, Changsha, China

**Keywords:** ETS family, melanoma, ETV7, immune microenvironment, CD8+ T cell, single-cell RNA sequencing

## Abstract

The ETS family modulates immune response and drug efficiency to targeted therapies, but their role in melanoma is largely unclear. In this study, the ETS family was systematically analyzed in multiple public data sets. Bioinformatics tools were used to characterize the function of ETV7 in melanoma. A prognostic model was constructed using the LASSO Cox regression method. We found that ETV7 was the only differentially expressed gene with significant prognostic relevance in melanoma. Enrichment analysis of seven independent data sets indicated ETV7 participation in various immune-related pathways. ETV7 particularly showed a strong positive correlation with CD8+ T cell infiltration. The prognostic model based on ETV7 and its hub genes showed a relatively good predictive value in training and testing data sets. Thus, ETV7 can potentially regulate the immune microenvironment in melanoma.

## Introduction

Melanoma, originating from pigment-producing melanocytes, comprises 75% of deaths related to skin cancer (1). In 2019, a total of 96,480 new cases of melanoma of the skin reported were in the United States (2). At its early stage, the disease could be surgically removed, with a relatively good prognosis; however, when it spreads to distant organs, the 5-year survival rate of patients with this disease sharply declines to roughly 10%, as indicated by a recent review (3). Targeted therapies and immune checkpoint inhibitors (ICIs) have revolutionized the treatment of metastatic melanoma. However, numerous problems still need to be addressed. For instance, current evidence suggests that 40 to 65% of patients with advanced-stage metastatic melanoma show minimal or no RECIST response to ICIs at the outset (*de novo* resistance), and 43% of responders acquire resistance by 3 y (4, 5). Moreover, melanoma patients receiving mitogen-activated protein kinase (MAPK)-targeted therapy also develop resistance, which leads to relapse (6). Several underlying mechanisms have been revealed, such as NRAS mutations and BRAF amplification, but 40% of them present unknown resistance mechanisms beyond genetic alterations (6–8).

Recent studies find that transcriptional factors (TFs) relevant to the MAPK pathways modulate drug efficiency to MAPK inhibitors (9, 10). For instance, overexpression of ETV1, ETV4, or ETV5 can sufficiently restore cell proliferation in the presence of trametinib, a MEK inhibitor (9). An early study also observes that overexpression of ETV1 confers resistance to MAPK inhibitors in BRAF-mutant melanoma (11). ETV1, ETV4, and ETV5 are members of the E26 transformation-specific or E-twenty-six (ETS) family, which is one of the largest transcriptional factor families and participates in multiple biological processes such as cellular differentiation, cell cycle control (12), cell migration (13), and cell proliferation (14). The family consists of 28 TFs in humans and binds to similar DNA sequences of MAPK (15). Various studies have revealed that aberrant expression of many ETS family members is strongly associated with tumor initiation, progression, and metastasis in cancer (16–18). Some ETS inhibitors, such as VPC-18005 and BRD32048, exert antitumor effects in pre-clinical studies (19, 20). In skin melanoma, the function of the ETS family is poorly studied. ETS1 can promote aggregation and invasion of melanoma (21, 22), and ETV2 is required during tumor angiogenesis (23); meanwhile, the role of the rest of the ETS family members is largely unclear.

In addition, the ETS family has been shown to play a role in immunity (24–26). ELF1, ETV4, ETV3L, ETS1, and ETS2 can up-regulate the expression of leukocyte-associated immunoglobulin-like receptor-1, which inhibits the maturation, differentiation, and activation of immune cells (25, 27). ELF4 can inhibit the proliferation of naive CD8+ T cells by increasing the expression of KLF4 (24, 28). However, the regulation of immune response in cancer by ETS family members, as well as the mechanism underlying this regulation, has yet to be determined.

In the current study, we systematically assessed the expression profile, prognostic significance, and role of ETS family members in human skin cutaneous melanoma (SKCM) by integrating data from The Cancer Genome Atlas (TCGA) database, the Genotype–Tissue Expression (GTEx) Project, Oncomine database, cBioPortal database, and Gene Expression Omnibus (GEO) database. Our results indicate that ETV7 is markedly downregulated in melanoma, and low ETV7 expression is related to the poor prognosis of SKCM patients. Enrichment analysis and immune profile analysis indicate that ETV7 may regulate the differentiation and activation of T cells. This finding suggests a previously unrecognized involvement of ETV7 in immunity and the potential of the gene to modulate the immune response to the disease.

## Materials and Methods

### Data Acquisition and Processing

**Figure 1:**
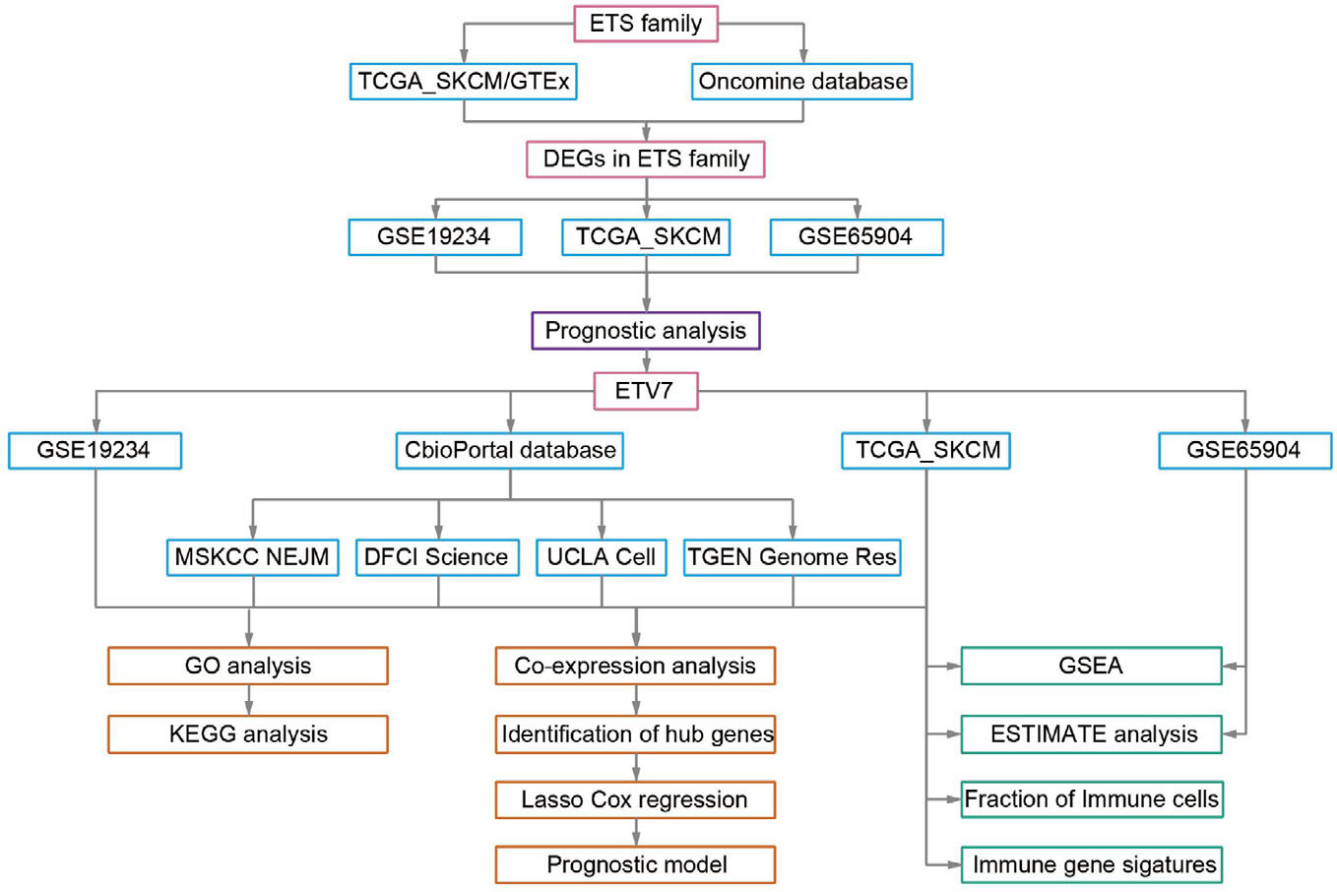
Workflow of this study.

The workflow of this study is presented in **Figure 1**. The TCGA_SKCM data set and GEO data sets (GSE65904 and GSE19234) were acquired and processed using the method described in our previous study (29). After data filtering was conducted, 448 tumor samples with survival data in the TCGA_SKCM data set, 210 tumor samples in GSE65904, and 44 samples in GSE19234 were used for further analysis. The single-cell RNA sequencing data (GSE72056) were downloaded and analyzed directly in the current study because the data had been processed and normalized (30). All data used in the present study were acquired from public databases, requiring no further approval by an ethics committee.

### Online Database Analysis

The Oncomine database (https://www.oncomine.org/resource/login.html) was used to validate the transcription level of the genes of interest in skin melanoma by retrieving expression data (log2-transformed) in three cohorts of melanoma *vs.* normal tissues for statistical comparison, with the following default thresholds: P-value < 1E-4, fold change >2, and gene ranks in the top 10%.

The GEPIA2 (http://gepia2.cancer-pku.cn/) database was also used to compare the transcript level of the genes of interest between melanoma tumors and normal samples from TCGA and GTEx projects.

The cBioPortal for Cancer Genomics (http://www.cbioportal.org/) was used to identify genes that exhibit a strong positive correlation (R > 0.5, q-value < 0.05) with ETV7 in all available melanoma-related data sets with gene expression data.

The human protein atlas (https://www.proteinatlas.org/) was used to analyze the expression of ETV7 across a set of normal human cells or tissues.

### Functional Analysis and Enrichment Analysis

The co-expressed genes of ETV7 shared in five independent data sets were constructed into a protein–protein interaction (PPI) network in the STRING database (http://string-db.org). Cytoscape version 3.7.2 was used to visualize these networks. Hub genes were identified as the top 10 nodes, based on the score generated by the cytoHubba plugin in Cytoscape (ranked by degree).

Gene ontology (GO) and Kyoto Encyclopedia of Genes and Genomes (KEGG) enrichment analyses were conducted using the clusterProfiler package in R version 3.6.2 (18).

Gene set enrichment analysis (GSEA) was used to investigate pathways enriched in the high- and low-ETV7 groups. *C2.cp.kegg.v7.1.symbols.gmt* was chosen as the gene set database. The pathways were considered significantly enriched with the following criteria: nominal p-value < 0.05, false discovery rate q-value < 0.25, and normalized enrichment score > 1.

### Immune Profile Analysis

The immune and stromal scores of each sample were estimated using the ESTIMATE algorithm in the ‘estimate’ package in R version 3.6.2 (31). Immune cell infiltration in each sample of the TCGA_SKCM data set was conducted by preparing and uploading gene expression data into the TIMER2.0 website (http://timer.cistrome.org/) in accordance with the instruction on the website (32).

### Construction of the Prognostic Model

The least absolute shrinkage and selection operator (LASSO) Cox regression analysis was conducted using the glmnet package in R. The analysis generated key gene signatures and their corresponding coefficients, which were used to calculate the novel score, as follows: score = −0.08241781*CCR5 − 0.01208383*IFNG − 0.13002695*TBX21 − 0.04765633*CXCL10 − 0.33444001*CXCR3-0.05630411*CCL5. To facilitate the interpretation of results from different data sets, the risk score was calculated by subtracting the minimum score of the cohort from this score, and dividing the difference by the absolute value of the maximum score of the cohort—that is, namely, risk score = (score − Min)/absolute(Max).

### Statistical Analysis

The data collected were analyzed by default as described using web resources. The remaining data were analyzed using R version 3.6.2. The median expression of genes or the median value of the risk score was used as cutoff value in dividing patients into two subgroups. Univariate Cox regression and multivariate Cox regression were conducted using the survminer package in R. The survival analyses were compared using the Kaplan–Meier method with the logrank test. Time-dependent receiver operator characteristic (ROC) analyses and subsequent calculation of the area under the curve (AUC) were performed using the timeROC package in R. Correlation analysis between the gene expression of ETV7 and that of the remaining genes was conducted by R software version 3.6.2 with spearman method. Wilcoxon test was conducted to compare gene expression between groups. Packages in R used for data analysis and graph plotting included ggplot2, ggpubr, limma, vennDiagram, tidyverse, rms, org.Hs.eg.db, dplyr, Rtsne, and plyr. P < 0.05 was considered statistically significant (*, P < 0.05; **, P < 0.01; ***, P < 0.001; ****, P < 0.0001).

## Results

### Identification of Differentially Expressed ETS Family Members in Melanoma

**Figure 2:**
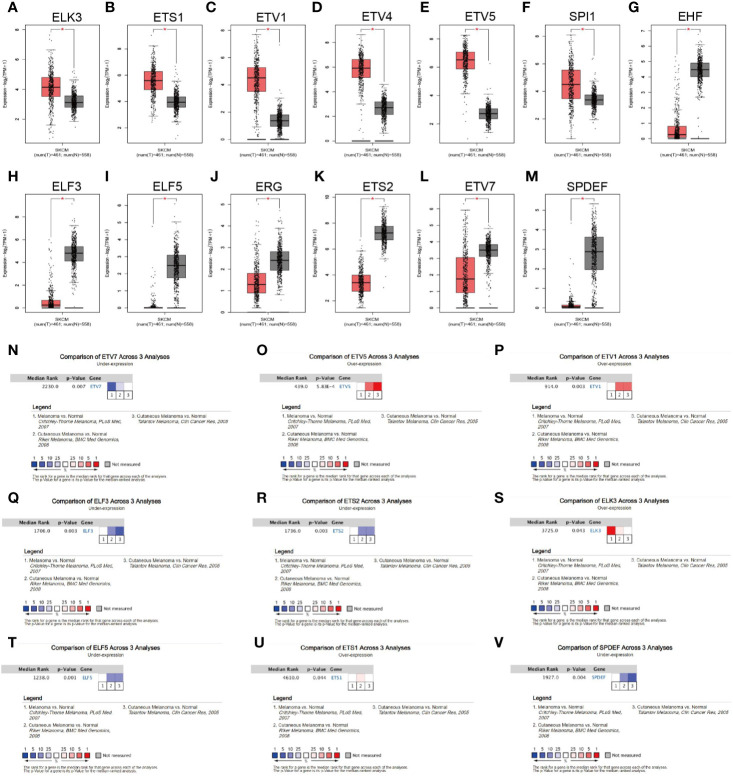
Expression profile of the ETS family in melanoma. **(A–M)** Expression levels of ELK3 **(A)**, ETS1 **(B)**, ETV1 **(C)**, ETV4 **(D)**, ETV5 **(E)**, SPI1 **(F)**, EHF **(G)**, ELF3 **(H)**, ELF5 **(I)**, ERG **(J)**, ETS2 **(K)**, ETV7 **(L)**, and SPDEF **(M)** in melanoma and corresponding normal tissues, based on the GEPIA2 database. **(N–V)**. Data from the Oncomine database verifying that ETV7 **(N)**, ETV5 **(O)**, ETV1 **(P)**, ELF3 **(Q)**, ETS2 **(R)**, ELK3 **(S)**, ELF5 **(T)**, ETS1 **(U)**, and SPDEF **(V)** were differentially expressed between tumor and normal tissues across three cohorts.

The TCGA_SKCM project has only one normal sample. Thus, we first investigated the expression of all ETS family members in melanoma and normal skin by manipulating the data from the GTEx database. GEPIA2 integrates gene expression data from the TCGA and GTEx projects *via* a standard processing pipeline (33). Using the default cutoff value of GEPIA2 (|Log2FC| cutoff = 1, and q-value cutoff = 0.01), we found that 13 of the 28 family members were differentially expressed (**Figures 2A–M**, **Supplementary Figures 1A–O**). In particular, ELK3, ETS1, ETV1, ETV4, ETV5, and SPI1 were significantly upregulated in melanoma (**Figures 2A–F**), whereas EHF, ELF3, ELF5, ERG, ETS2, ETV7, and SPDEF were significantly downregulated in the tumor (**Figures 2G–M**). Further, the expression profiles of ETV1, ETV5, ETV7, ELF3, ETS1, ETS2, ELK3, ELF5, and SPDEF were validated by comparing their transcriptional level between melanoma and normal tissues across three independent cohorts from the Oncomine database (**Figures 2N–V**). However, the remaining members were not differentially expressed between tumor and normal tissues (**Supplementary Figures 1P–S**).

### ETV7 as an Independent Prognostic Predictor in Melanoma

**Figure 3:**
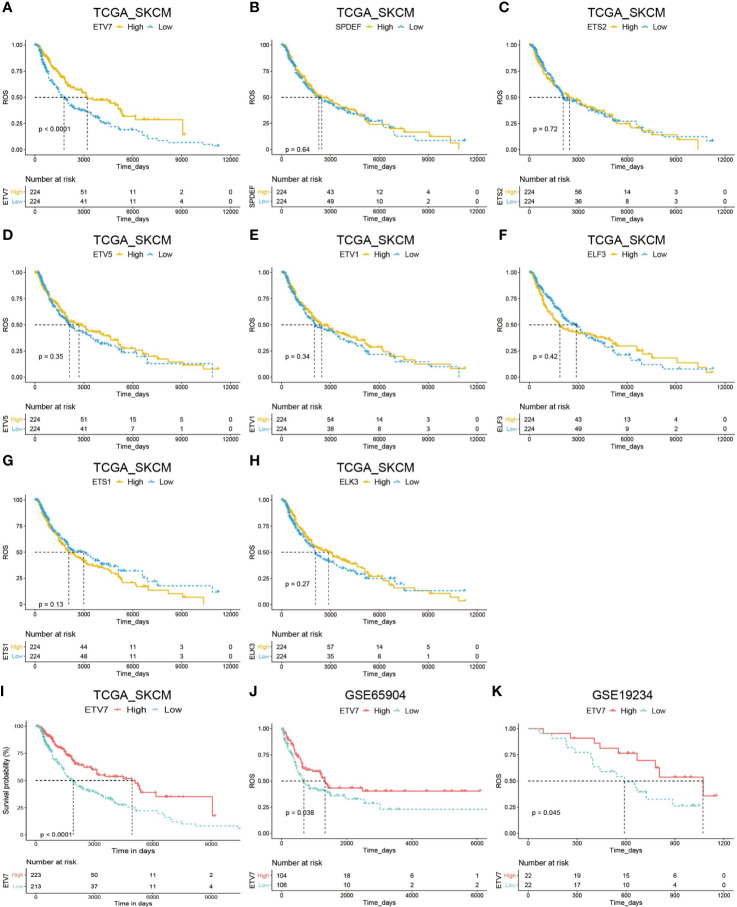
Prognostic analysis of DEGs of the ETS family in melanoma. **(A–H)** Kaplan–Meier plot of overall survival (OS) in melanoma patients in the TCGA_SKCM cohort with high and low expression of ETV7 **(A)**, SPDEF **(B)**, ETS2 **(C)**, ETV5 **(D)**, ETV1 **(E)**, ELF3 **(F)**, ETS1 **(G)**, and ELK3 **(H)**. **(I)** Kaplan–Meier plot of DDS of melanoma patients in the TCGA_SKCM cohort with high and low expression of ETV7. **(J)** Kaplan–Meier plot of DDS of melanoma patients in the GSE65904 cohort with high and low expression of ETV7. **(K)** Kaplan–Meier plot of survival time since metastasis of melanoma patients in the GSE19234 cohort with high and low expression of ETV7.

We subsequently evaluated the prognostic significance of the above nine differentially expressed ETS family members in TCGA_SKCM. Analysis suggested that EVT7 exhibited prognostic significance in melanoma patients (**Figures 3A–H**). Moreover, melanoma patients with high ETV7 showed significantly longer disease-specific survival (DSS) than those with low ETV7 (p < 0.0001, **Figure 3I**). We further evaluated the prognostic relevance of these nine differentially expressed genes (DEGs) in the GSE65904 data set and found a similar result for ETV7 (p = 0.038, **Figure 3J**). By contrast, the remaining genes showed no prognostic significance (**Supplementary Figures 2A–H**). In addition, data from the GSE19234 data set showed that melanoma patients with low ETV7 had significantly shorter survival time since metastasis (p = 0.045, **Figure 3K**).

As shown in **Table 1**, univariate Cox regression analysis indicated that low ETV7 (p < 0.001), age > 60 y (p < 0.001), Breslow depth >2 cm (p < 0.001), Clark level IV–V (p < 0.001), non-White patients (p = 0.004), advanced stage (III–IV, p < 0.001), advanced T stage (T3–Tx, p < 0.001), and advanced N stage (N1–Nx, p < 0.001) are associated with shorter OS. In addition to the M stage, these factors underwent multivariate Cox regression analysis. The result showed that ETV7 and the N stage remained to be independent risk factors (**Table 1**).

**Table 1:** Univariate and multivariate Cox regression analyses of ETV7 and clinicopathologic features in melanoma patients.

Clinicopathologic variable	Univariate cox analysis		Multivariate cox analysis	
	HR (95% CI)	p-value	HR (95% CI)	p-value
ETV7 (low *vs.* high)	1.709 (1.303–2.242)	<0.001	1.529 (1.072–2.182)	0.019
Age (<60 *vs.* ≥60)	0.619 (0.469–0.915)	<0.001	0.721 (0.507–1.024)	0.068
Breslow depth (<2 mm *vs.* ≥2 mm)	0.449 (0.327–0.615)	<0.001	0.498(0.223–1.113)	0.089
Clark level (I–III *vs.* IV–V)	0.488 (0.344–0.693)	<0.001	0.755 (0.499–1.144)	0.186
Race (White *vs.* non-White)	0.349 (0.171–0.711)	0.004	0.684 (0.093–5.027)	0.709
Stage (0–II *vs.* III–IV)	0.589 (0.441–0.785)	<0.001	1.290 (0.678–2.452)	0.438
T (T0–T2 *vs.* T3–TX)	0.583 (0.435–0.782)	<0.001	1.282 (0.559–2.939)	0.558
N (N0 *vs.* N1–NX)	0.604 (0.458–0.796)	<0.001	0.370 (0.198–0.691)	0.002
M (M0 *vs.* M1/MX)	0.582 (0.307–1.103)	0.097	0.533 (0.189–1.506)	0.235
Gender (male *vs.* female)	1.134 (0.855–1.503)	0.383	–	–
Multiple primary tumors present (No *vs.* YES)	0.647 (0.342–1.224)	0.181	–	–
Prior systemic therapy (No *vs.* YES)	1.356 (0.826–2.228)	0.229	–	–
Radiation therapy (No *vs.* YES)	1.753 (0.836–3.678)	0.138	–	–
Prior malignancy diagnoses (No *vs.* YES)	0.851 (0.485–1.493)	0.573	–	–

**Figure 4:**
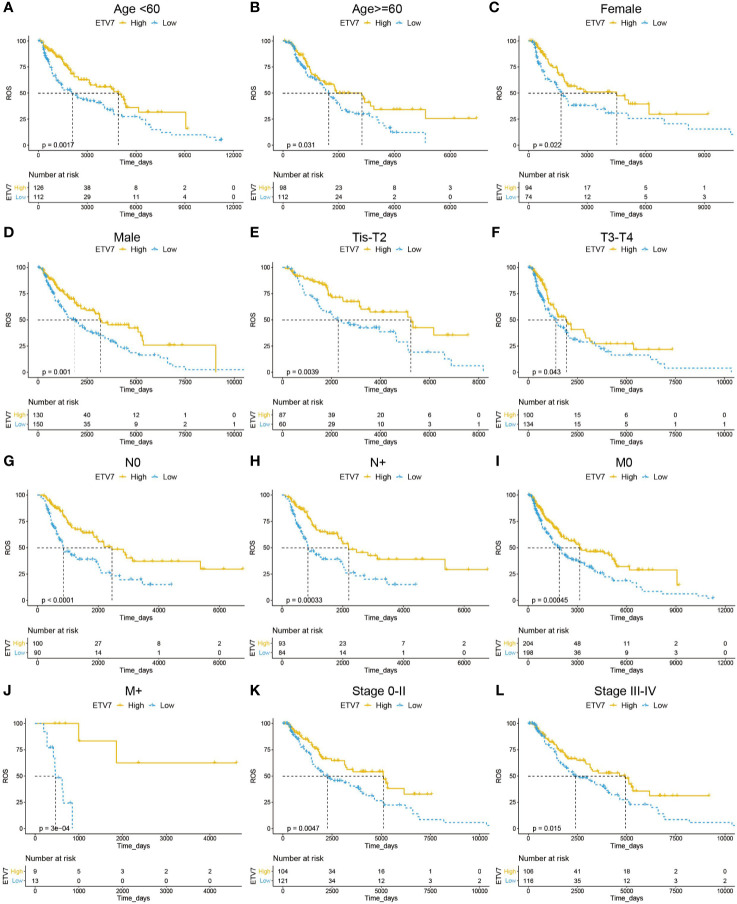
Prognostic analysis of ETV7 in different subgroups of melanoma patients. **(A, B)** Kaplan–Meier plot of OS in high and low-ETV7 subgroups of melanoma patients < 60 y **(A)** or older than 60 y **(B)**. **(C, D)** Kaplan–Meier plot of overall survival (OS) in female **(C)** or male **(D)** melanoma patients with high and low expression of ETV7. **(E, F)** Kaplan–Meier plot of OS in high and low-ETV7 subgroups of melanoma patients at early (Tis–T2, **E**) or advanced (T3–T4, **F**) T stage. **(G, H)** Kaplan–Meier plot of OS in high and low-ETV7 subgroups of melanoma patients with **(G)** or without **(H)** lymph node metastasis. **(I, J)** Kaplan–Meier plot of OS in high- and low-ETV7 subgroups of melanoma patients with **(I)** or without **(J)** distant metastasis. **(K, L)** Kaplan–Meier plot of OS in high- and low-ETV7 subgroups of melanoma patients at the early **(K)** or advanced **(L)** stage.

We then investigated the prognostic relevance of ETV7 in different subgroups of melanoma patients. As shown in **Figures 4A, B**, low ETV7 expression was correlated with a significantly short OS in melanoma patients younger than 60 y (**Figure 4A**, p = 0.0017) or older than 60 y (**Figure 4B**, p=0.031). A similar association between ETV7 and survival time was also observed in both male and female patients (**Figures 4C, D**), patients with different T, N, and M stages (**Figures 4E, F**), and patients with melanoma at the early (**Figure 4K**, p = 0.0047) or advanced (**Figure 4L**, p = 0.015) stage.

### Coexpression and Enrichment Analysis

**Figure 5:**
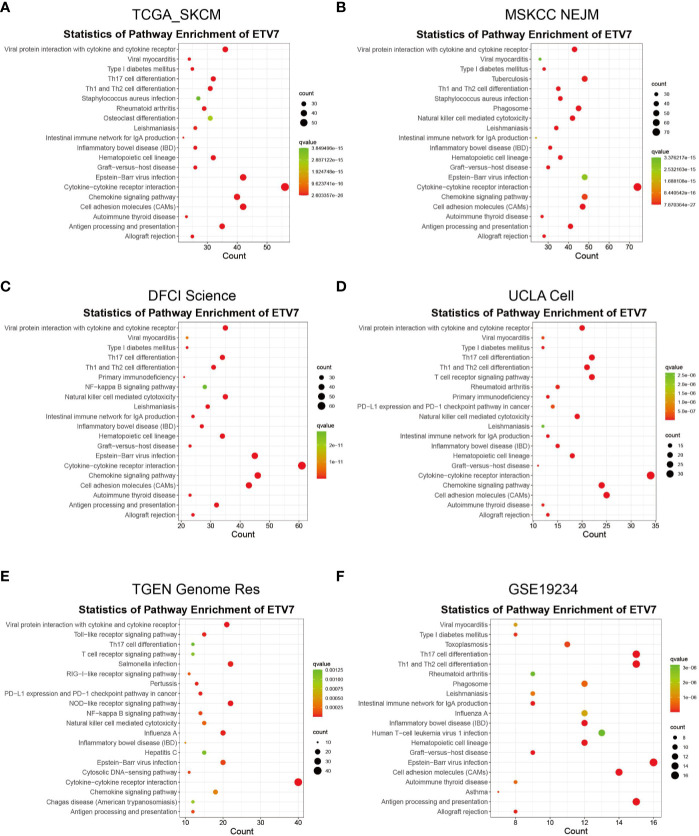
KEGG analysis of ETV7-related genes in TCGA_SKCM **(A)**, MSKCC NEJM **(B)**, DFCI Science **(C)**, UCLA Cell **(D)**, TGEN Genome Res **(E)**, and GSE19234 **(F)** data sets.

The aforementioned analysis indicated that ETV7 was significantly downregulated in melanoma and was associated with the prognosis of melanoma patients. To understand the function of ETV7 in melanoma, we first identified the ETV7related genes, which were defined as those exhibiting strong correlation (coefficient >= 0.5, p < 0.05) with ETV7 at the transcriptional level in melanoma. A total of 623 ETV7-related genes were found in the TCGA_SKCM data set (**Supplementary Table 1**). GO analysis indicated that these genes were enriched in immune-related biological processes and molecular functions such as T cell activation, regulation of T cell activation, regulation of lymphocyte activation, response to interferon-gamma, regulation of leukocyte proliferation, MHC protein binding, cytokine receptor activity, chemokine receptor binding, MHC class II receptor activity, and CCR chemokine receptor binding (**Supplementary Figure 3A**). KEGG analysis suggested that these genes were enriched in antigen processing and presentation, cell adhesion molecules, cytokine–cytokine receptor interaction, Th1 and Th2 cell differentiation, Th17 cell differentiation, chemokine signaling pathway, natural killer (NK) cell-mediated cytotoxicity, and PD-L1 expression and PD-1 checkpoint pathway in cancer (**Figure 5A**, **Supplementary Table 2**).

We further searched the cBioportal database and found that in addition to the TCGA_SKCM data set, four of nine melanoma-related data sets contained gene expression data: *MSKCC NEJM*, *DFCI Science*, *UCLA Cell*, and *TGEN Genome Res*. Coexpression analysis of ETV7 in these four data sets identified 1 021 ETV7-related genes in the *MSKCC NEJM* cohort, 981 genes in the *DFCI Science* cohort, 440 genes in the *UCLA Cell* cohort, and 667 genes in the *TGEN Genome Res* cohort. The GO and KEGG analyses of these genes in each cohort consistently indicated that they were enriched in similar immune-related processes or pathways (**Figures 5B–E**, **Supplementary Figures 3B–E**). The coexpression and subsequent GO and KEGG analyses of GSE19234 further verified the association between ETV7 and immune-related pathways (**Figure 5F**, **Supplementary Figure 3F**).

**Figure 6:**
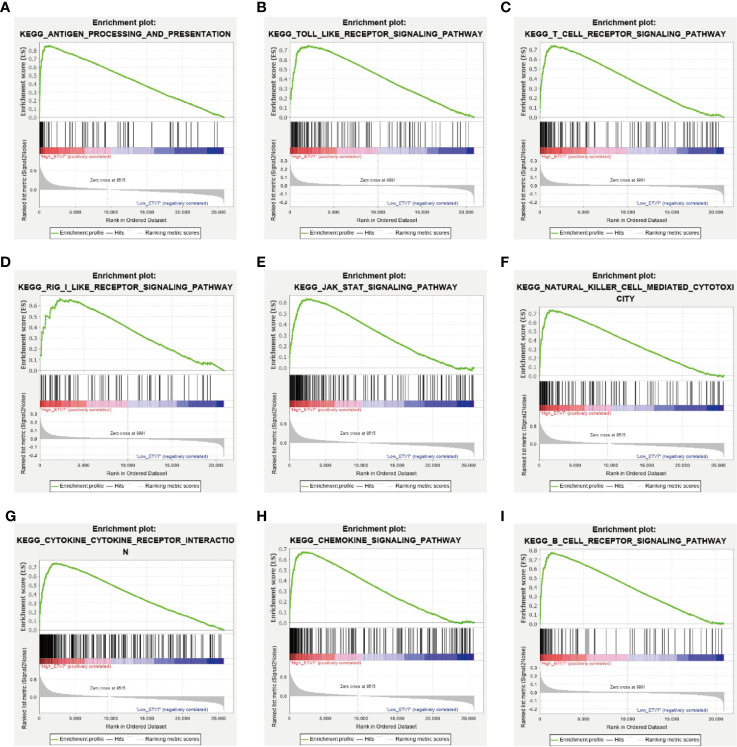
GSEA between melanoma patients with high and low ETV7 expression in the TCGA_SKCM and GSE65904 cohorts **(A–I)**.

In addition, GSEA was applied to investigate the difference in signaling pathways between the low and high ETV7 expression subgroups in the TCGA_SKCM and GSE65904 data sets. The results inferred that various immune-related pathways were enriched in the high ETV7 expression subgroups. These pathways included antigen processing and presentation, Toll-like receptor signaling pathway, T cell receptor signaling pathway, RIG-I like receptor signaling pathway, JAK-STAT signaling pathway, B cell receptor signaling pathway, NK cell-mediated cytotoxicity, cytokine–cytokine receptor interaction, and Fc gamma R-mediated phagocytosis (**Figures 6A–I**, **Supplementary Table 3**).

### Immune Profile

**Figure 7:**
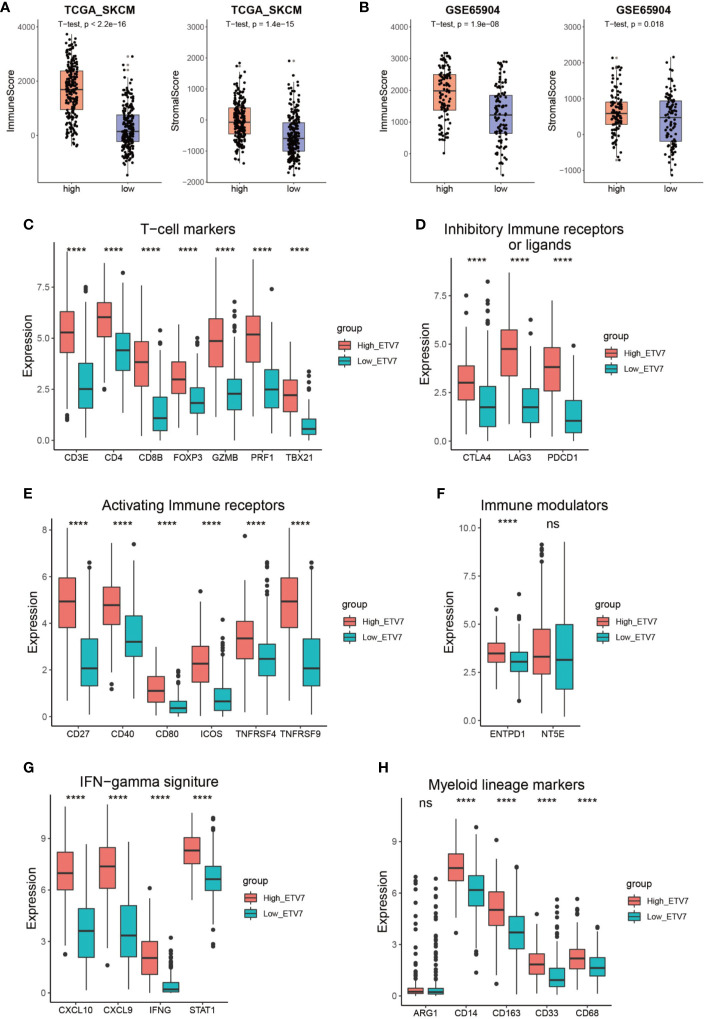
Immune profile of melanoma patients with high and low ETV7 expression. **(A, B)** Immune and stromal scores of melanoma patients with high and low ETV7 expression in the TCGA_SKCM **(A)** and GSE65904 **(B)** cohorts. **(C–H)** Comparison of the expression of T cell markers **(C)**, inhibitory immune receptors or ligands **(D)**, activating immune receptors **(E)**, immune modulators **(F)**, IFN*γ* signatures **(G)**, and myeloid lineage phenotypic and functional markers **(H)** between melanoma patients with high and low ETV7 expression. ****: p < 0.0001. ns, no significant.

**Figure 8:**
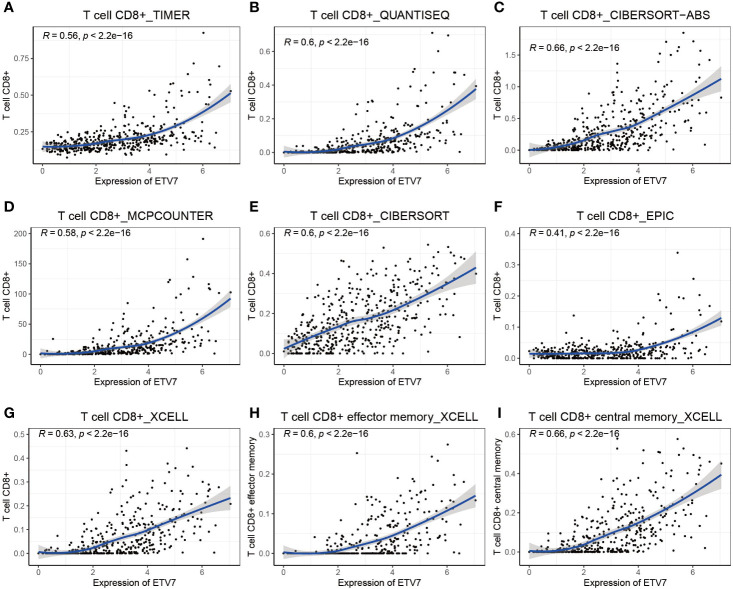
Correlation between the expression of ETV7 and abundance of CD8+ T cells **(A–I)**.

The aforementioned analysis suggests a close link between ETV7 and immune-related pathways. We thus employed the ESTIMATE method to evaluate the overall TME status in the high- and low-ETV7 subgroups of melanoma patients. In the TCGA_SKCM and GSE65904 cohorts, the immune and stromal scores were significantly higher in the high-ETV7 subgroups than in the low-ETV7 subgroups (**Figures 7A, B**). All phenotypic and functional markers of T cells—CD3E, CD4, CD8B, FOXP3, GZMB, PRF1 and TBX21—were expressed at higher levels in melanoma patients with high ETV7 expression (**Figure 7C**). Notably, the inhibitory immune receptors or ligands (CTLA4, LAG3, and PDCD1) and activating immune receptors (CD27, CD40, CD80, ICOS, TNFRSF4, and TNFRSF9) were significantly elevated in patients with high ETV7 expression, indicating a complex immune response in this subgroup (**Figures 7D, E**). Among the immune modulators, ENTPD1 was highly expressed in the high-ETV7 subgroup of melanoma patients, whereas NT5E showed no significant difference (**Figure 8F**). Moreover, IFN*γ* signatures (CXCL10, CXCL9, IDO1, IFNG, and STAT1) and myeloid lineage phenotypic and functional markers (CD14, CD163, CD33, and CD68) were also significantly increased in melanoma patients with high ETV7 (**Figures 7G, H**). However, ARG1, a previously reported M2 macrophage marker, was not differently expressed in the two subgroups (**Figure 7H**).

Further, multiple immune deconvolution methods—namely, TIMER, QUANTISEQ, CIBERSORT, MCPCOUNTER, XCELL, and EPIC—were manipulated to estimate the abundances of immune cells infiltrating into melanoma (34, 35). Analyzing the relationship between ETV7 and the abundance of different immune cells, we observed a strong positive correlation between ETV7 and CD8+ T cells, as determined by TIM (**Figure 8A**, r = 0.56, p < 2.2*e^−16^), QUANTISEQ (**Figure 8B**, r = 0.6, p < 2.2*e^−16^), CIBERSORT (**Figure 8C**, r = 0.6, p < 2.2*e^−16^), CIBERSORT-ABS (**Figure 8D**, r = 0.66, p < 2.2*e^−16^), MCPCOUNTER (**Figure 8E**, r = 0.58, p < 2.2*e^−16^), EPIC (**Figure 8F**, r = 0.41, p < 2.2*e^−16^), and XCELL (**Figure 8G**, r = 0.63, p < 2.2*e^−16^). In addition, ETV7 exhibited a strong positive correlation with CD8+ effector-memory T cells (**Figure 8H**, r = 0.6, p < 2.2*e^−16^) and CD8+ central memory T cells (**Figure 8I**, r = 0.66, p < 2.2*e^−16^).

**Figure 9:**
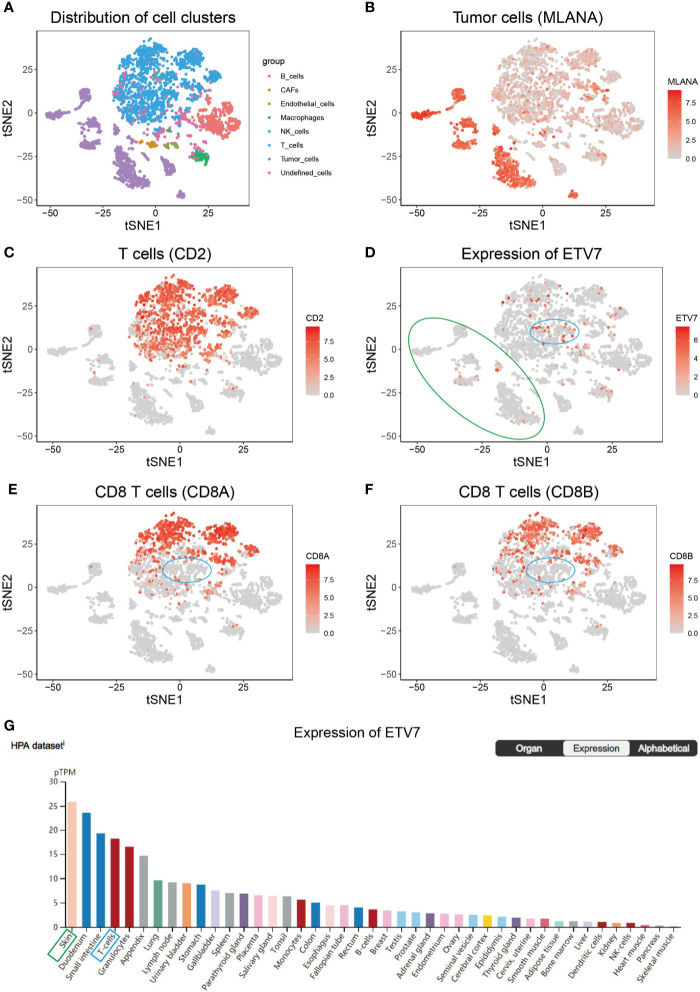
Single-cell RNA sequencing (scRNA-seq) analysis of ETV7 in melanoma. **(A)** t-Distributed stochastic neighbor embedding (t-SNE) analysis of different clusters of cells in the GSE72056 data set. **(B)** The average expression of MLANA for tumor cells overlaid on the tSNE plot. **(C)** Average expression of CD2 for T cells overlaid on the tSNE plot. **(D)** Average expression of ETV7 for cells overlaid on the tSNE plot. **(E)** Average expression of CD8A for CD8+ T cells overlaid on the tSNE plot. **(F)** Average expression of CD8B for CD8+ T cells overlaid on the tSNE plot. **(G)** Expression of ETV7 across a set of normal human cells. (A green circle denotes cluster of tumor cells; a blue circle indicates a specific fraction of T cells).

Previous studies indicate that ETV7 is predominantly expressed in hematopoietic tissues (36, 37); thus, the strong positive correlation between ETV7 and CD8+ T cell infiltration may be attributed to ETV7 expression in this subgroup of T cells. To test this hypothesis, we analyzed the GSE72056 data set, a public single-cell RNA sequencing (scRNA-seq) data set of melanoma patients (30). In this data set, the tumor cells were designated by copy number variation analysis, whereas the non-malignant cells were annotated as six types of cells—T cells, B cells, macrophages, endothelial cells, cancer-associated fibroblasts, and NK cells—based on preferentially or uniquely expressed marker genes (30). The remaining unresolved cells were referred to as undefined cells in the current study. Consistent with the original study by Itay Tirosh et al. (30). non-linear dimensionality reduction (t-distributed stochastic neighbor embedding (t-SNE)) analysis revealed that these eight clusters of cells could be distinguished separately (**Figure 9A**). Tumor cells exhibited markedly elevated expression of MLANA, a widely used biomarker of melanoma (**Figure 9B**). CD2 was exclusively expressed in T cells (**Figure 9C**), macrophages had distinct CD163 expression (**Supplementary Figure 4A**), and B cells were marked by CD19 expression (**Supplementary Figure 4B**) (30). As shown in **Figure 9D**, ETV7 was not exclusively expressed in T cells but could be detected in several types of cells, including tumor cells (green circle), T cells, and macrophages. ETV7 was also not detected in most CT8+ T cells (labeled as CD8A+ and/or CD8B+, **Figures 9D–F**), and a negligible fraction of ETV7-positive T cells (**Figures 9D–F**, blue circle) were not recognized as CD8+ T cells. Collectively, these results indicate that the strong positive correlation between ETV7 and infiltration of CD8+ T cells cannot be attributed to ETV7 expression in these cells. In addition, we found that skin (green block, **Figure 9G**) showed the highest ETV7 expression across a set of normal human cells or tissues, including T cells (denoted by a blue block, **Figure 9G**, **Supplementary Figure 4C**).

### Construction of an ETV7-Related Prognostic Model

**Figure 10:**
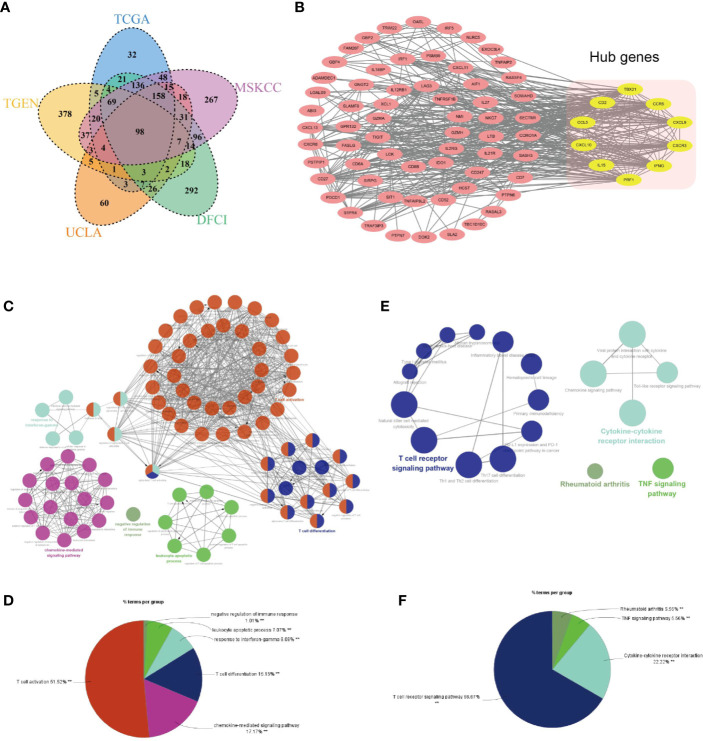
Identification of hub genes of ETV7-related genes. **(A)** A Venn diagram of ETV7-related genes of the five data sets. **(B)** A protein–protein interaction network constructed using STRING and visualized with Cytoscape (yellow modules denote hub genes). **(C, D)** Biological process analysis of the 98 ETV7-related genes by using the ClueGo plugin in the Cytoscape. **(E, F)** KEGG analysis of the 98 ETV7-related genes by using the ClueGo plugin in Cytoscape.

As shown in **Figure 10A**, 98 genes are commonly shared among the ETV7-related genes in the five aforementioned data sets. A PPI network was constructed in the STRING database and visualized in Cytoscape (**Figure 10B**). Consistent with the results of GO, KEGG, and GSEA analyses, ClueGo analysis demonstrated that these 98 genes were predominantly involved in immune-related biological processes such as T cell activation, chemokine-mediated signaling pathway, T cell differentiation, and response to interferon-gamma (**Figures 10C, D****)**, as well as in immune-related pathways such as the T cell receptor signaling pathway, cytokine–cytokine receptor interaction, and TNF signaling pathway (**Figures 10E, F**) (38). The 10 hub genes were identified as CCR5, IFNG, TBX21, CXCL10, PRF1, CD2, CXCR3, CXCL9, CCL5, IL15 (**Figure 10B**). All melanoma patients exhibiting low expression of these hub genes showed a significantly shorter OS (**Supplementary Figures 5A–J**).

**Figure 11:**
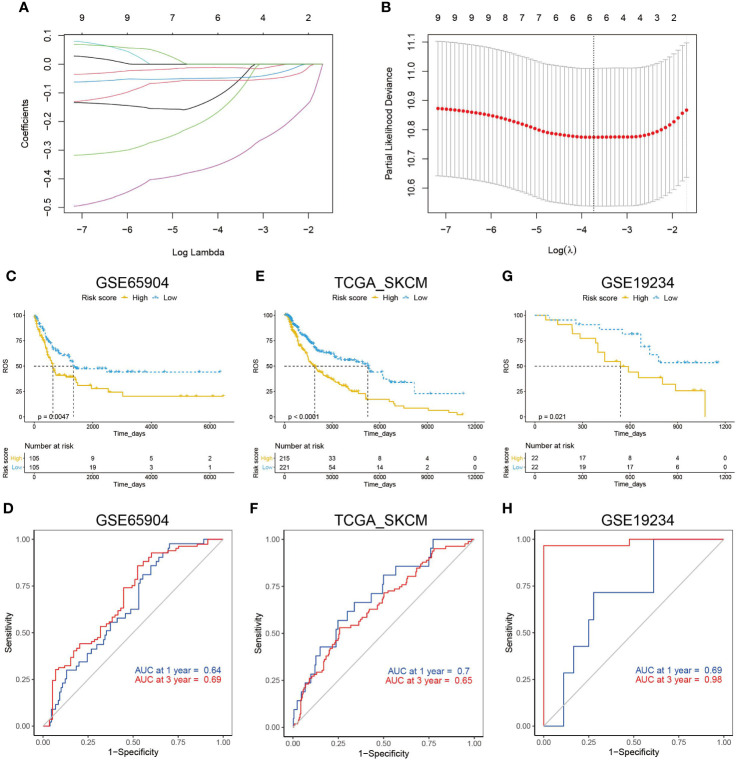
Construction of an ETV7-related prognostic model. **(A, B)** A LASSO Cox regression model constructed from ETV7 and the 10 hub genes, with the tuning parameter (*λ*) calculated based on partial likelihood deviance with tenfold cross-validation. An optimal log *λ* value shown by the vertical black line in the plot. **(C)** Kaplan–Meier plot of DDS in the high- and low-risk groups of the GSE65904 cohort. **(D)** Time-dependent ROC analysis of the risk score for DDS and survival status in the GSE65904 cohort. **(E)** Kaplan–Meier plot of DDS in the high- and low-risk groups of the TCGA_SKCM cohort. **(F)** Time-dependent ROC analysis of the risk score for DDS and survival status in the TCGA_SKCM cohort. **(G)** Kaplan–Meier plot of survival time since metastasis in the high- and low-risk groups of the GSE19234 cohort. **(H)** Time-dependent ROC analysis of the risk score for survival time since metastasis and survival status in the GSE19234 cohort.

To construct an ETV7-related prognostic model for the disease-specific survival of melanoma patients, ETV7 and 10 hub genes were input into a LASSO Cox regression model in the GSE65904 data set (n = 210). Six genes were selected using the partial likelihood deviance method (**Figures 11A, B**). These genes were CCR5, IFNG, TBX21, CXCL10, CXCR3, and CCL5. The risk score was calculated by inputting the selected signature genes into the aforementioned formula. The median value of the risk score was set as the cutoff value, dividing patients into low- and high-risk subgroups. Prognostic analysis with the Kaplan–Meier method showed that melanoma patients with low risk scores had a significantly longer DSS (**Figure 11C**, p = 0.0047) in the GSE65904 cohort. Time-dependent ROC analysis in this cohort demonstrated that the risk score had a favorable predictive value (**Figure 11D**, AUC at 1 year = 0.64, AUC at 3 y = 0.69). The prognostic value of the established risk score was validated in the TCGA cohort (**Figure 11E**, p < 0.0001). The ROC analysis in this data set showed that the AUC at 1 y reached 0.7 and at 3 y was 0.65 (**Figure 11F**). Further, melanoma patients with high risk scores showed significantly shorter survival times since metastasis than those with low risk scores in the GSE19234 cohort (p = 0.021, **Figure 11G**). The ROC analysis in this data set indicated that the AUC at 1 y reached 0.69 and at 3 y was 0.98 (**Figure 11H**).

## Discussion

In this systematic analysis of the ETS family in melanoma, we first integrated the data from the TCGA and GTEx databases and three available cohorts of melanoma patients in the Oncomine database; we then found that ELK3, ETS1, ETV1, and ETV5 were significantly upregulated in melanoma, whereas ELF3, ELF5, ETS2, ETV7, and SPDEF were significantly downregulated in the tumor. Prognostic analysis of these DEGs in the TCGA_SKCM and GSE65904 data sets consistently showed that only ETV7 had significant prognostic relevance in melanoma (**Figures 3A, J**). Moreover, the gene could serve as an independent prognostic predictor after correcting for other confounding factors (**Table 1**). Indeed, all patients in various subgroups of melanoma with low ETV7 expression showed a significantly shorter OS than those with high ETV7 expression (**Figure 4**). Several studies reported that ETS1 supported cell growth (39), prevented apoptosis (40), and facilitated the invasion of melanoma (22); however, an early study demonstrated that the gene was widely expressed in benign and malignant melanocytes, and immunohistochemical analysis indicated that its expression had no significant association with the clinical outcome (41), supporting the results of the current study. Two studies also observed that 5.3–18% of melanoma patients exhibited an increase in ETV1 expression. Overexpression of ETV1, combined with oncogenic NRAS (G12D), can transform primary melanocytes and promote tumor formation in mice (42, 43). Although ETV1 showed no significant prognostic relevance in melanoma in the present study, its importance in a subset of melanomas with ETV1 amplification could not be underestimated and ignored. In addition, no studies have reported on the expression and function of ELK3, ETV5 ELF3, ELF5, ETS2, and SPDEF in melanoma. Although in this study, their expression was not correlated with prognosis, their involvement in the development of certain cases of melanoma could not be excluded, and future research has to be conducted.

As a member of the ETS transcription factor family, ETV7 in melanoma has not been previously investigated. Our knowledge of the gene under physiological and pathological conditions is considerably limited. Initially identified at the beginning of this century, ETV7 was found to be highly related to the oncogenic ETV6 (36). However, recent studies have found that the two genes exerted opposite biological effects, and ETV7 can inhibit the transcriptional activities of ETV6 (44, 45). ETV7 has earlier been reported to act as a hematopoietic oncoprotein; however, three recent studies found that the gene could suppress the proliferation, migration, and invasion of some solid tumors such as oral squamous cell carcinoma and nasopharyngeal carcinoma (45–49). To explore the role of ETV7 in melanoma, we analyzed seven independent melanoma-related data sets (TCGA_SKCM, GSE65904, GSE19234, and four data sets from the cBioportal database) by conducting GSEA, GO, and KEGG analyses, which are widely used bioinformatics tools in the functional characterization of specific genes (50–53). All of these enrichment analyses suggest broad ETV7 involvement in immune-related processes and pathways, including the following: T cell activation; Th1, Th2, and Th17 cell differentiation; PD-L1 expression and PD-1 checkpoint pathway in cancer; and antigen processing and presentation (**Figures 5****–****7**). Melanoma patients with high ETV7 expression had significantly higher immune scores than those with low ETV7, suggesting higher immune cell infiltration in the tumor microenvironment. Indeed, when the infiltration of various immune cells in melanoma was estimated, ETV7 showed a strong positive correlation with the infiltration of CD8+ T cells (**Figure 9**). Moreover, melanoma patients with high ETV7 exhibited a significantly higher expression of various T cell markers, such as CD3E, CD4, CD8B, and TBX21 (54); this finding verified the close link between ETV7 and T cell infiltration. With the ETV7 expression in hematopoietic cells considered, the strong correlation between ETV7 and the infiltration of CD8+ T cells may be attributed to ETV7 expression in T cells rather than tumor cells. Thus, we first evaluated ETV7 expression across a set of normal human cells or tissues in the human protein atlas; the skin was found to exhibit the highest ETV7 expression. We then analyzed the public scRNA-seq data set (GSE72056) and found that most of the CD8+ T cells showed no ETV7 expression, and some ETV7-positive T cells were not CD8+ T cells (**Figures 9C–F**). ETV7 could be detected in some melanoma tumor cells (**Figure 9D**). ETV was highly expressed in normal skin and showed markedly decreased expression in melanoma (**Figures 2L, N** and **9G**); thus, not all melanoma cells might be expected to express ETV7 (**Figure 9D**).

Moreover, early studies inferred a positive regulatory role of ETV7 in T and B cells (45, 48). In the study by Cintia Carella et al., all mice that received transplants with TEL2-expressing bone marrow died from T-cell lymphoma, whereas none of the control mice developed hematopoietic malignancy with the chemical carcinogen N-ethyl-N-nitrosourea (45). Monica Cardone et al. showed that ETV7 could inhibit apoptosis and promote B cell proliferation by targeting the cell cycle and apoptotic regulators (48). The results obtained from the present study, combined with other studies, highly suggest that ETV7 can have an essential role in T cell differentiation, proliferation, infiltration, and activation in melanoma.

Notably, several members of the ETS family regulate the immune system (55, 56). ETS1 regulates the differentiation of several types of immune cells, such as T helper cell subsets and cytotoxic T cells; it also directly controls the expression of cytokine and chemokine genes (57). The mechanism by which ETV7, a member of the ETS family, regulates the immune process and response in melanoma has yet to be determined. However, 10 hub genes were identified in the current study (**Figure 10B**). Among them, CD2 is a transmembrane glycoprotein typically known for its participation in the costimulatory pathway of T cell activation (58). TBX21 is essential for naive T lymphocyte development and interferon-gamma production (59). IL15 is identified as a cytokine that stimulates the proliferation of T lymphocytes (60). PRF1 encodes the protein perforin, which is present in T and NK cells and facilitates the release of granzymes and subsequent cytolysis of target cells (61). CCR5, CXCL10, CXCR3, CXCL9, CCL5, and IFNG also participate in the migration, trafficking, or differentiation of T cells (61–65). Whether ETV7 binds the promoter of these genes and stimulates their expression remains unknown, but our analysis showed a high correlation between them and ETV7 at the transcriptional level. To clarify this concern, future studies have to be conducted.

We further constructed a prognostic model by using LASSO Cox regression, a broadly selected machine learning algorithm to minimize the risk of overfitting (66). The model had a relatively good predictive value in training and testing cohorts (**Figures 11D, F**). All six gene signatures were immune-related and regulated the proliferation, differentiation, migration, or activation of T cells (59, 60, 62, 63, 65). In addition, melanoma patients with low expression of these six gene signatures had a significantly shorter OS (**Supplementary Figure 5**). T cells, particularly CD8+ T cells, demonstrate a predominant role in the anticancer effects of ICIs. Thus, the model can potentially help select patients who can be responsive to ICIs; however, this hypothesis should be tested by prospective analysis in multicenter cohorts.

## Conclusions

A systematic analysis of the ETS family in melanoma identified an essential role of ETV7 in regulating the immune microenvironment of the disease. Further studies are recommended to explore the exact mechanism that allows ETV7 to regulate the proliferation, migration, infiltration, and activation of immune cells, particularly CD8+ T cells.

## Data Availability Statement

The original contributions presented in the study are included in the article/**Supplementary Material**. Further inquiries can be directed to the corresponding author.

## Author Contributions

HQ and HZ contributed equally to this work. The study was conceived and designed by ZS. Analysis of data was performed by HQ, HZ, and ZS. Bioinformatics analysis in R was conducted by ZS and XZ. Useful discussion and advice were provided by YL, FL, and LS. ZS drafted the article. All authors contributed to the article and approved the submitted version.

## Funding

This work is supported by the New Xiangya Talent Project of the Third Xiangya Hospital of Central South University (Grant No. JY201715).

## Conflict of Interest

The authors declare that the research was conducted in the absence of any commercial or financial relationships that could be construed as a potential conflict of interest.

## Supplementary Material

Supplementary Figure 1 Expression profile of the ETS family in melanoma. **(A–O)** Expression of ELF1 **(A)**, SPIC **(B)**, SPIB **(C)**, GABPA **(D)**, FLI1 **(E)**, ETV3L **(F)**, ETV3**(G)**, ETV2 **(H)**, FEV **(I)**, ETV6 **(J)**, ERF **(K)**, ELK4 **(L)**, ELK1 **(M)**, ELF4 **(N)**, and ELF2 **(O)** in melanoma and corresponding normal tissues, based on the GEPIA2 database. **(P–S)**. Data from the Oncomine database show that EHF **(P)**, SPI1 **(Q)**, ETV4 **(R)**, and ERG **(S)** are not differentially expressed between tumor and normal tissues across three cohorts.

Supplementary Figure 2 Prognostic analysis of the differentially expressed genes of the ETS family in melanoma. **(A–H)** Kaplan–Meier plot of overall survival in melanoma patients in the GSE65904 cohort with high and low expression of ELF3 **(A)**, SPDEF **(B)**, ETV5 **(C)**, ETV1 **(D)**, ETS2 **(E)**, ETS1 **(F)**, ELK3 **(G)**, or ELF5 **(H)**.

Supplementary Figure 3 GO enrichment analysis of ETV7-related genes in the TCGA_SKCM **(A)**, MSKCC NEJM **(B)**, DFCI Science **(C)**, UCLA Cell **(D)**, TGEN Genome Res **(E)**, and GSE19234 **(F)** data sets.

Supplementary Figure 4 Single-cell RNA sequencing (scRNA-seq) analysis in melanoma. **(A)** Average expression of CD163 for macrophages overlaid on the tSNE plot. **(B)** Average expression of CD19 for B cells overlaid on the tSNE plot. **(C)** Expression of ETV7 across a set of normal human tissues.

Supplementary Figure 5 Prognostic analysis of ETV7-related hub genes in melanoma. **(A–J)** Kaplan–Meier plot of overall survival of melanoma patients in the TCGA_SKCM cohort with high and low expression of CCL5 **(A)**, TBX21 **(B)**, PRF1 **(C)**, IL15 **(D)**, IFNG **(E)**, CXCR3 **(F)**, CXCL10 **(G)**, CXCL9 **(H)**, CD2 **(I)**, or CCR5 **(J)**.
