# Supplementary material for: Integrated Analysis of the ETS Family in Melanoma Reveals a Regulatory Role of ETV7 in the Immune Microenvironment
Source: Front Immunol. 2020 Dec 23;11:612784. doi: 10.3389/fimmu.2020.612784 (PMC7786291; doi:10.3389/fimmu.2020.612784)

Supplementary Figure 1


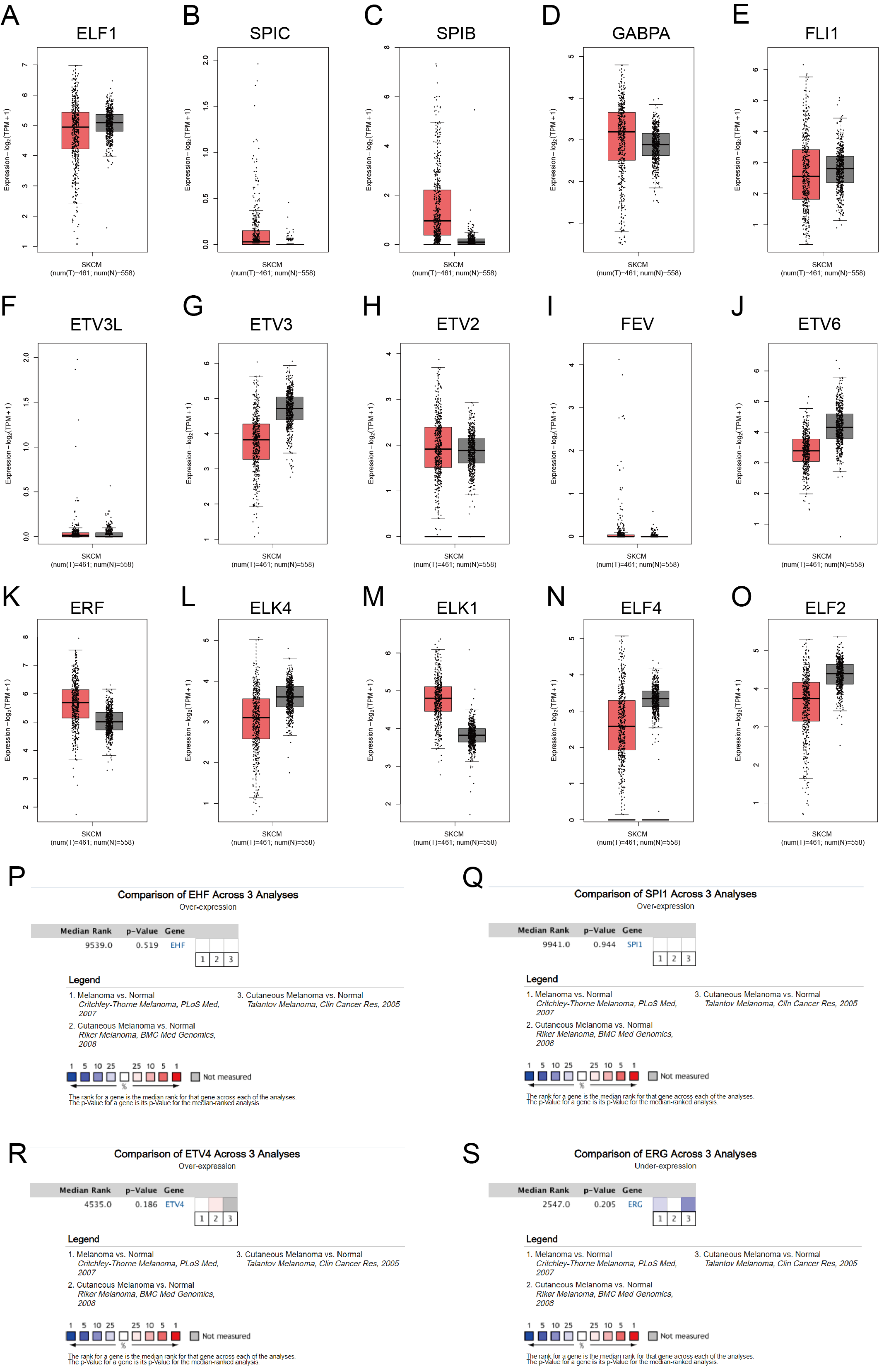


Supplementary Figure 2


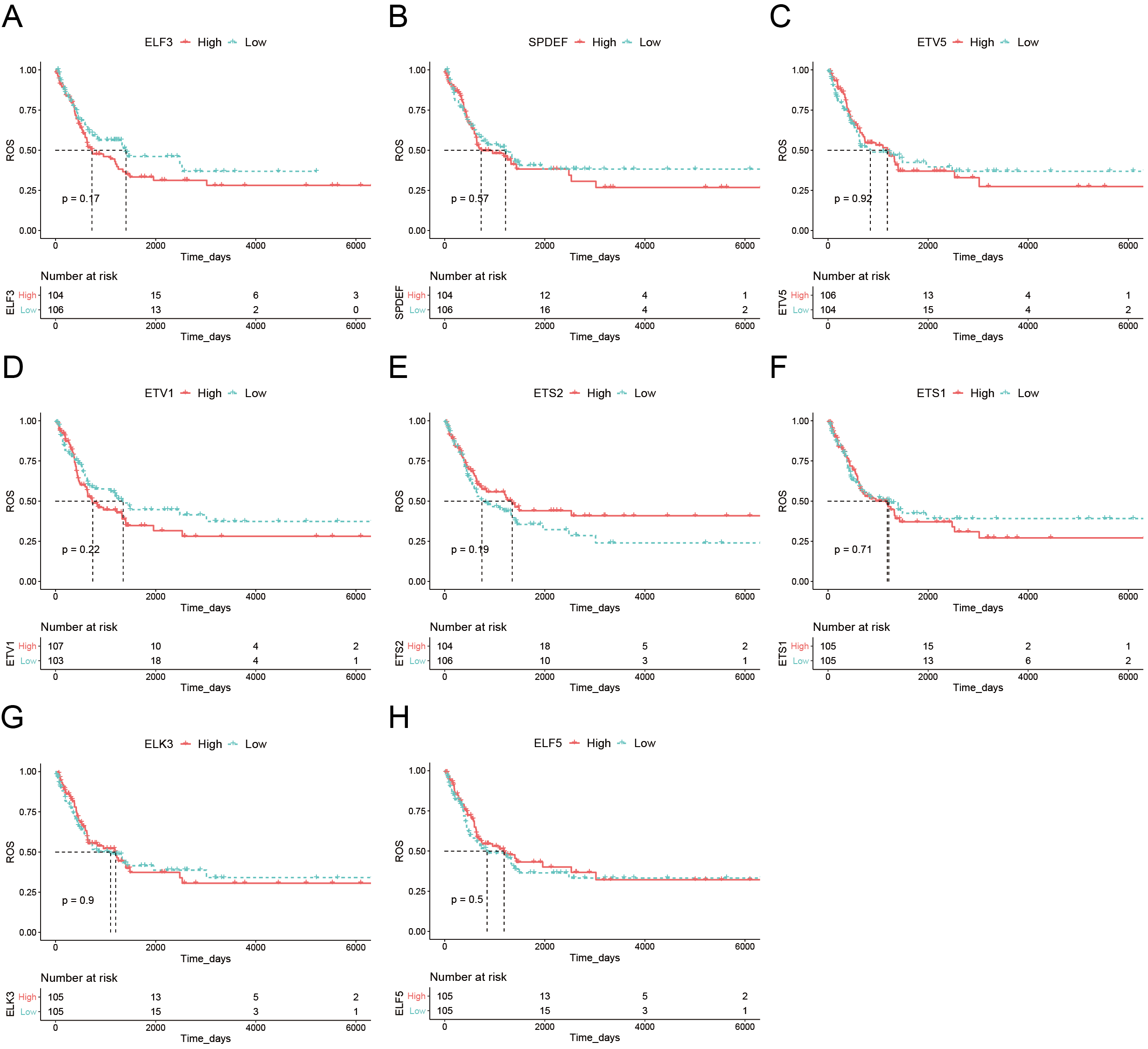


Supplementary Figure 3


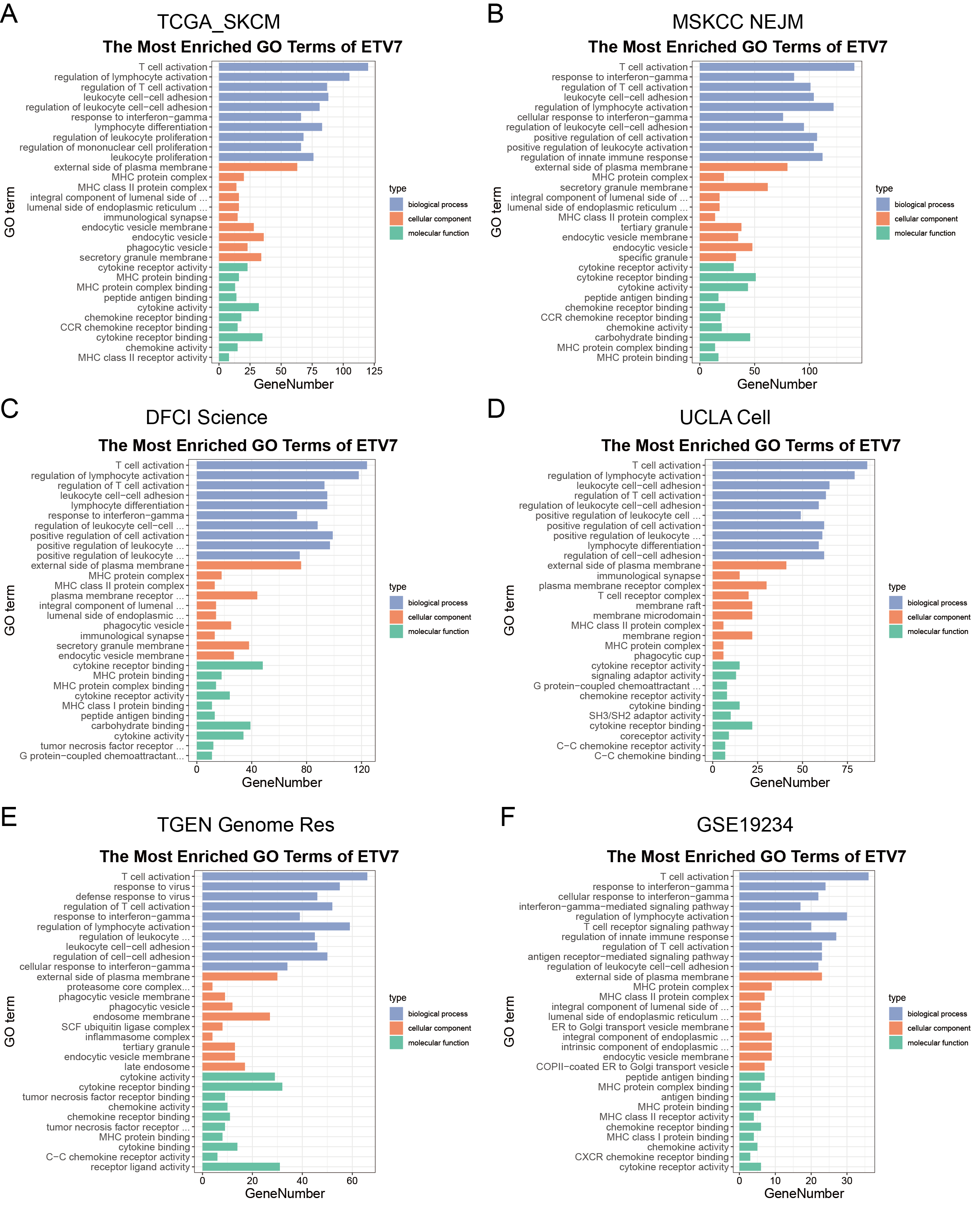


Supplementary Figure 4


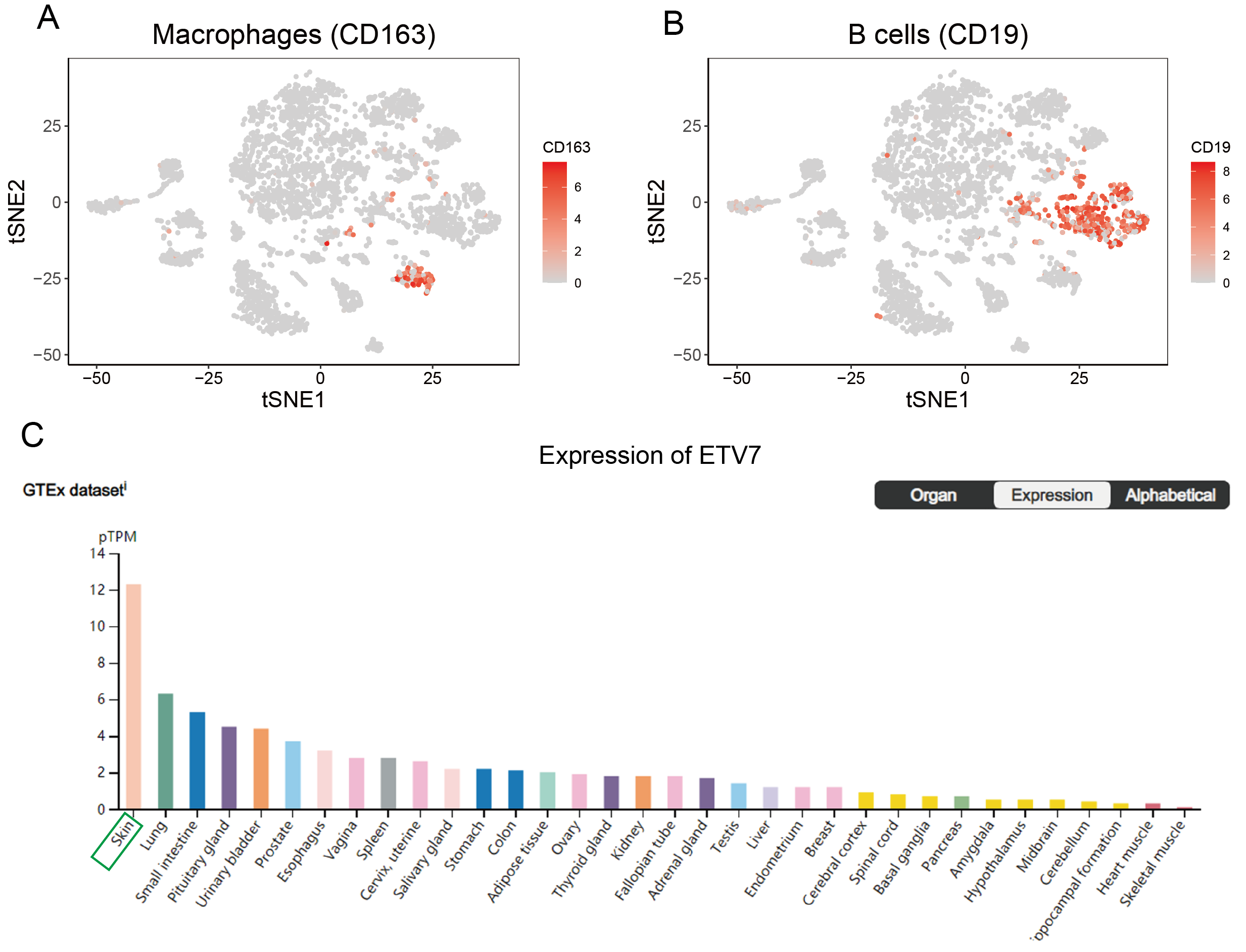


Supplementary Figure 5


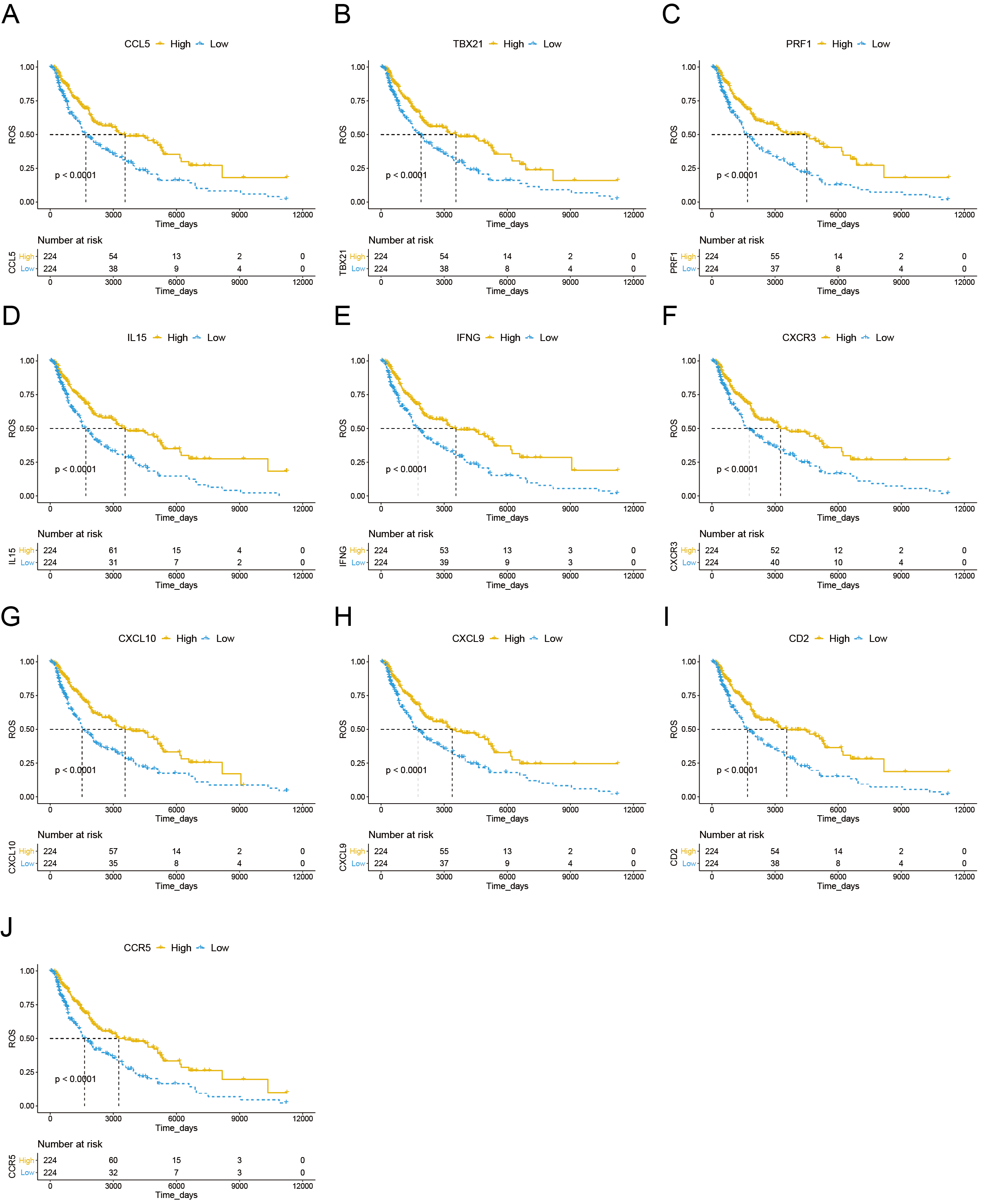

Supplement: Supplementary Figure 1 — Expression profile of the ETS family in melanoma. (A–O) Expression of ELF1 (A), SPIC (B), SPIB (C), GABPA (D), FLI1 (E), ETV3L (F), ETV3(G), ETV2 (H), FEV (I), ETV6 (J), ERF (K), ELK4 (L), ELK1 (M), ELF4 (N), and ELF2 (O) in melanoma and corresponding normal tissues, based on the GEPIA2 database. (P–S). Data from the Oncomine database show that EHF (P), SPI1 (Q), ETV4 (R), and ERG (S) are not differentially expressed between tumor and normal tissues across three cohorts. [file DataSheet_1.docx]
